# MicroRNA-181c inhibits cigarette smoke–induced chronic obstructive pulmonary disease by regulating CCN1 expression

**DOI:** 10.1186/s12931-017-0639-1

**Published:** 2017-08-15

**Authors:** Yong Du, Yi Ding, Xuru Chen, Zhoufang Mei, Heyuan Ding, Yi Wu, Zhijun Jie

**Affiliations:** 1 Department of Respiratory Medicine, The Fifth People’s Hospital of Shanghai, Fudan University, No.801 Heqing Road, MinhangDistrict, Shanghai, 200240 China; 2 Department of Endocrinology, The Fifth People’s Hospital of Shanghai, Fudan University, No.801 Heqing Road, MinhangDistrict, Shanghai, 200240 China

**Keywords:** miR-181c, COPD, CCN1, Lung injury, Inflammatory cytokines

## Abstract

**Background:**

Chronic obstructive pulmonary disease (COPD) is an obstinate pulmonary disease, causing irreversible alveoli collapse and increasing the risk for cardiovascular disease. Accumulating evidence has shown that the dysregulation of miRNAs is crucially involved in the pathogenesis and development of COPD. However, the effects and role of microRNA-181c (miR-181c) have not been investigated in a murine model of COPD.

**Methods:**

miR-181c expression was detected in human lung tissue samples of 34 patients, an in vivo murine model of CS exposure, and primary human bronchial epithelial cells (HBECs) by qRT-PCR. Degeneration of lung tissue, necrosis, infiltration and neutrophil cells were assessed with H&E and flow cytometry. Interleukin (IL)-6 and IL-8 levels were determined by an enzyme-linked immunosorbent assay and qRT-PCR. Luciferase reporter assay and correlation analyses were used to confirm and measure the levels between miR-181c and its target CCN1.

**Results:**

We showed that miR-181c was significantly down-regulated in lung tissues from patients with COPD compared to individuals who had never smoked (*p* < 0.01). We also observed a down-regulation of miR-181c in HBECs and a mouse model after cigarette smoke (CS) exposure. Functional assays demonstrated that miR-181c over-expression decreased the inflammatory response, neutrophil infiltration, reactive oxygen species (ROS) generation, and inflammatory cytokines induced by CS, while its down-regulation produced the opposite effects. Subsequent investigation found that CCN1 was a direct target of miR-181c. CCN1 expression was increased in lung tissues of COPD patients, and was negatively correlated with miR-181c expression in human COPD samples (*p* < 0.01).

**Conclusions:**

Taken together, our data suggest the critical roles of miR-181c and its target CCN1 in COPD development, and provide potential therapeutic targets for COPD treatment.

## Background

Chronic obstructive pulmonary disease (COPD) is a common, preventable and treatable disease that is characterized by persistent respiratory symptoms and a progressive airflow limitation due to airway and/or alveolar abnormalities usually caused by an abnormal inflammatory response of the lung to noxious particles and gases [1]. COPD increases the risk for cardiovascular disease and is currently the fourth leading cause of death worldwide [2]. According to the WHO Global Alliance Against Chronic Respiratory Diseases, patients with COPD numbered approximately 329 million in 2010, along with 2.9 million deaths [3]. Cigarette smoking is by far the most important risk factor for COPD. Although smoking cessation is currently the only effective treatment for COPD, it only partially attenuates the accelerated decline in lung function. Thus, an improved understanding of the molecular mechanism underlying the development and progression of COPD is urgently needed to optimize strategies for more effective therapies.

MicroRNAs (miRNAs) are evolutionarily endogenous and conserved non-coding RNAs that play crucial regulatory roles in repressing protein expression by causing target mRNA degradation through binding to the 3′-untranslated region (3′-UTR) in a sequence-specific manner [4, 5]. Accumulating evidence has shown that the dysregulation of miRNAs is involved in the pathogenesis and development of COPD [6, 7]. Conickx and colleagues demonstrated that miR-218-5p plays a protective role in cigarette smoke–induced inflammation and COPD, indicating a crucial role for miR-218-5p in the pathogenesis of COPD [8]. miR-145 negatively regulates pro-inflammatory cytokine release from arterial smooth muscle (ASM) cells in COPD by targeting SMAD3 [9]. Cao et al. have reported that miR-183 might play a role in the expression of BKCaβ1, and that the expression of miR-183 and BKCaβ1 may be related to the pathogenetic pathways of COPD [10]. miR-181c is a member of the miR-181 family and plays an important role in inflammatory response, energy metabolism, and cancer development [11]. miR-181c has also been shown to be down-regulated in patients with COPD compared to never smokers [8]. However, no study has been conducted to investigate its role and mechanism in COPD.

In the present study, we found that miR-181c over-expression alleviated lung injury in COPD, and decreased the inflammatory response, neutrophil infiltration, and ROS generation, whereas miR-181c inhibition showed the opposite effect on COPD development. In addition, CCN1 was identified as the direct target of miR-181c in COPD. Taken together, our data suggest the critical roles of miR-181c and its target CCN1 in COPD development, and provide potential therapeutic targets for COPD treatment.

## Methods

### Human lung tissue and HBECs

Lung resection specimens were obtained from 34 patients at The Fifth People’s Hospital of Shanghai, of which 32 were from surgeries for solitary pulmonary tumors and 2 were from explant lungs of end-stage COPD patients undergoing lung transplantation (Table 1). Prior written informed consent and Institutional Ethics Committee approval were obtained for the use of these clinical materials for research purposes. The lung resection specimens 3 anonymous never somkers were digested by enzymes to obtain primary HBECs. HBECs were cultured in a high concentration of retinoic acid to enhance mucociliary differentiation for 14 days. Subsequently, HBECs were exposed to 2.5% cigarette smoke extract (CSE) for 24 h [8, 12]. The cells were harvested for RNA isolation and miR-181c expression was analyzed.

**Table 1:** Characteristics of study subjects

	Never smokers	Smokers	COPD
Number	8	8	18
Gender (male/female)	4/4	7/1	16/2
Age (years)	63 (58–74)	66 (56–72)	67 (59–71)
BMI	28 (24–31)	27 (22–30)	21 (17–25)
Current-smoker/ex-smoker	NA	6/2	11/7
Smoking history (pack-years)	0	38 (23–45)	50 (32–61)
FEV_1_ post-bronchodilator (L)	2.5 (2.1–2.7)	2.4 (1.8–2.5)	1.8 (1.5–2.2)
FEV_1_ post-bronchodilator (%predicted)	98 (85–108)	92 (82–101)	68 (60–77)
FEV_1_/FVC post-bronchodilator (%)	84 (75–88)	76 (70–79)	54 (51–61)
ICS (yes/no)	0/8	0/8	9/9

### Experimental procedures

All procedures for animal use were approved by the Animal Research Committee at Fudan University. Animal experiments were performed in accordance with the established International Guiding Principles for Animal Research [13]. C57BL/6 mice (male, 20–25 g) were fed under a 12 h light/dark cycle at 21–24 °C in the Animal Housing Unit. Standard laboratory chow and water were provided ad libitum. Animals were randomly assigned to control or experimental groups and allowed to acclimatize for at least 1 week before experimental procedures.

### Cigarette smoke extracts (CSE)

To prepare CSE, the smoke of 10 3R4F reference cigarettes was bubbled through 30 mL of RPMI 1640. The resulting suspension was filter-sterilized and defined as 100%. The CSE solution was diluted with RPMI 1640 to a final concentration of 2.5% and stored at 80 °C until use.

### Cigarette smoke (CS) exposure

Mice were exposed whole body to CS, as described previously [14]. Briefly, the animals were exposed to the tobacco smoke of five cigarettes, 4 times per day for 30 min. The animals were exposed 5 days per week for 24 weeks. As controls, C57BL/6 mice were exposed to room air. All the following pathophysiological evaluations were performed at 24 weeks after cigarette smoke exposure.

### Animal treatments

Animal experiments were performed in accordance with the established International Guiding Principles for Animal Research. miR-181c was over-expressed or inhibited using specific agomiRs and antagomiRs, respectively, which were synthesized by RiboBio Co. For in vivo over-expression or knockdown experiments, mice were intranasally injected with agomiR-181c or atagomiR-181c (10 nmol per mouse; *n* = 6 per group). AgomiR-181c, antagomiR-181c, a scrambled control, or PBS (solvent) was intranasally administered once every two weeks. The day after the last air or CS exposure, the mice were sacrificed and examined.

### Bronchoalveolar lavage

Lung tissues were lavaged with PBS after opening the thorax and exposing the trachea. 500 μL PBS was injected into and retrieved from the trachea twice, and this process was repeated 3 times for each mouse. The fluid was centrifuged at 1500 × *g* for 10 min. The supernatant was stored and used for determination of cytokine concentration. Cytokine concentration was determined using enzymatic-linked immunosorbent assay (ELISA) with antibodies from R&D Systems.

### RNA isolation and quantitative real-time PCR (qRT-PCR)

Total mRNA was isolated from 100 mg pulmonary tissues using the TRIzol reagent (Invitrogen) according to the manufacturer’s protocol. A total of 500 ng of RNA was used to synthesize the complementary DNA using a Prime Script 1st Strand cDNA Synthesis Kit (TaKaRa, Dalian, China) with microRNA specific RT-primers or oligo (dT)18 primers. The expression levels of miR-181c and CCN1 were detected using SYBR Premix Ex Taq (TaKaRa) according to the manufacturer’s instructions. CCN1 expression was determined using the primers 5′-AACCCGGATTTGTGAGGTGC-3′ (forward) and 5′-GCAGGAACCGCAGTACTTGG-3′ (reverse). Levels of miR-181c and mRNAs were normalized to RNU6B small nuclear RNA and β-actin, respectively, to yield a 2^-ΔΔCT^ value for relative expression of each transcript. All of the reactions were run in triplicate.

### Western blotting

Cells were isolated from lung tissue and lysed in RIPA buffer with Protease Inhibitor Cocktail. The protein content of lysates was measured using a BCA Protein Assay Kit. Proteins (40 μg) were electrophoresed in a 10% SDS–PAGE gel and transferred onto polyvinylidene fluoride membranes. The membranes were blocked with 5% milk for 1 h at room temperature, and incubated with primary antibody against CCN1 (Abcam) at 4 °C overnight. Then, the membranes were incubated with corresponding horseradish peroxidase (HRP)-conjugated secondary antibody at room temperature for 1 h. Signals were detected after chemiluminescent reaction with HRP Substrate. The protein expression level of GAPDH was used as a control.

### Vector construction and luciferase reporter assay

The human CCN1–3′-UTR target sequence (WT) was amplified from human genomic DNA. A sequence with a mutation in the miR-181c target site (MUT) was synthesized. The WT and MUT sequences were cloned into the pGL3-luciferase reporter vector, followed by sequencing verification. The luciferase reporter assay was performed as previously described [15]. Briefly, CCN1–3′-UTR-WT or CCN1–3′-UTR-MUT reporter vector, and agomiR-181c or scramble control were trasfected into cells using Lipofectamine 2000 (Invitrogen). The Dual-GLO Luciferase Assay System (Promega, Madison, WI) was used to calculate the relative luciferase activity (the ratio of Firefly/Renilla luminescence) according to the manufacturer’s protocol. For each plasmid construct, the transfection experiments were performed in triplicate.

### Measurement of ROS

CM-H2DCFDA (Molecular Probes, Carlsbad, CA), a cell-permeable dye, was used to detect ROS production [16]. Briefly, a total of 200 μL of cell suspension (10^5^cells/ml) was seeded into a 96-well plate in the presence of CM-H2DCFDA and incubated for 45 min at 37 °C. The signal intensity was analyzed using a fluorescence plate reader. ROS production was calculated based on the H_2_O_2_ standard curve. The results were averaged among three independent experiments.

### Statistical analysis

GraphPad Prism Software (GraphPad Software, San Diego, CA, USA) was used for statistical analysis. The data are expressed as the mean ± SD from at least three separate experiments. The relationship between miR-181c expression and CCN1 mRNA expression levels was analyzed using Pearson correlation analysis. The statistical significance was determined using non-parametric tests (Kruskall-Wallis; Mann-Whitney U). *p* < 0.05 was considered significant.

## Results

### Levels of miR-181c expression in lung tissues of COPD patients and CS-exposed mice

**Fig. 1:**
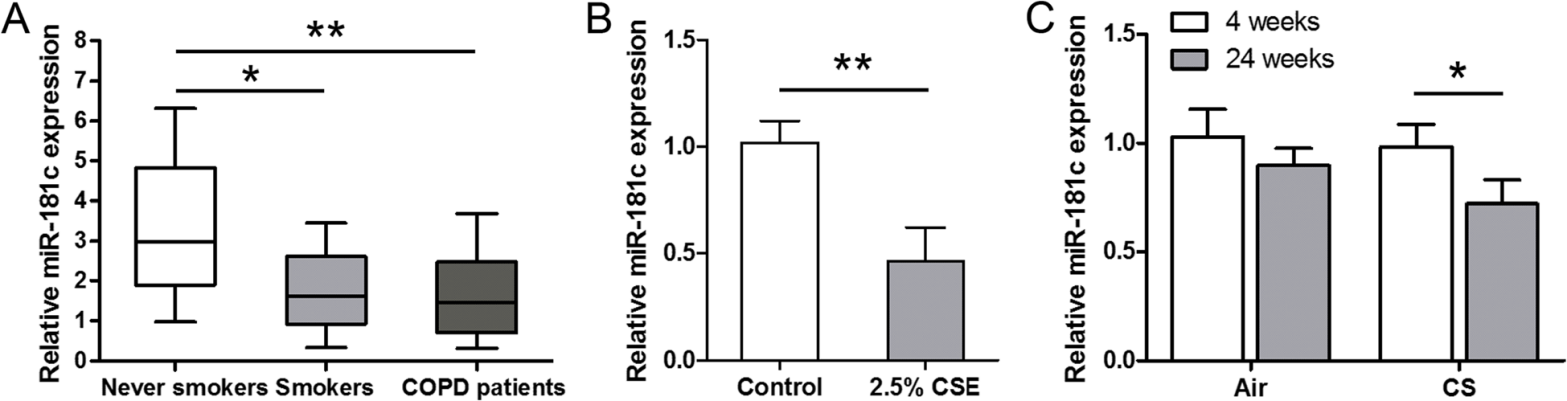
miR-181c expression was decreased in the lung tissues of COPD patients and CS-exposed mice. **a** The relative expression levels of miR-181c were examined by qRT-PCR in lung tissues of 8 never smokers, 8 smokers without COPD and 18 COPD patients. **b** The expression levels of miR-181c in HBECs 24 h after CSE exposure. **c** miR-181c expression was tested in lungs of mice exposed to air or CS for 4 or 24 weeks. *p < 0.05, **p < 0.01

We first determined the expression pattern of miR-181c in a total of 34 human lung tissue samples, including 8 never smokers, 8 smokers without COPD and 18 COPD patients. Compared with never smokers, the relative expression levels of miR-181c were significantly decreased in lung tissues of smokers and COPD patients (*p* < 0.01; Fig. 1a). In addition, we exposed HBECs to 2.5% CSE or control medium, and found that the expression of miR-181c in CSE-treated cells was significantly decreased by 53% as compared with control cells (*p* < 0.01; Fig. 1b). We also analyzed the miR-181c expression in a mouse model of CS exposure. Consistent with our observations in the human lung, miR-181c was significantly down-regulated in lung tissues of mice exposed to CS for 24 weeks, compared with air-exposed mice (*p* < 0.05; Fig. 1c). These data indicated that miR-181c was decreased in COPD and may serve as an inhibitor of COPD.

### In vivo administration of agomiR-181c or antagomiR-181c in CS-exposed mice

**Fig. 2:**
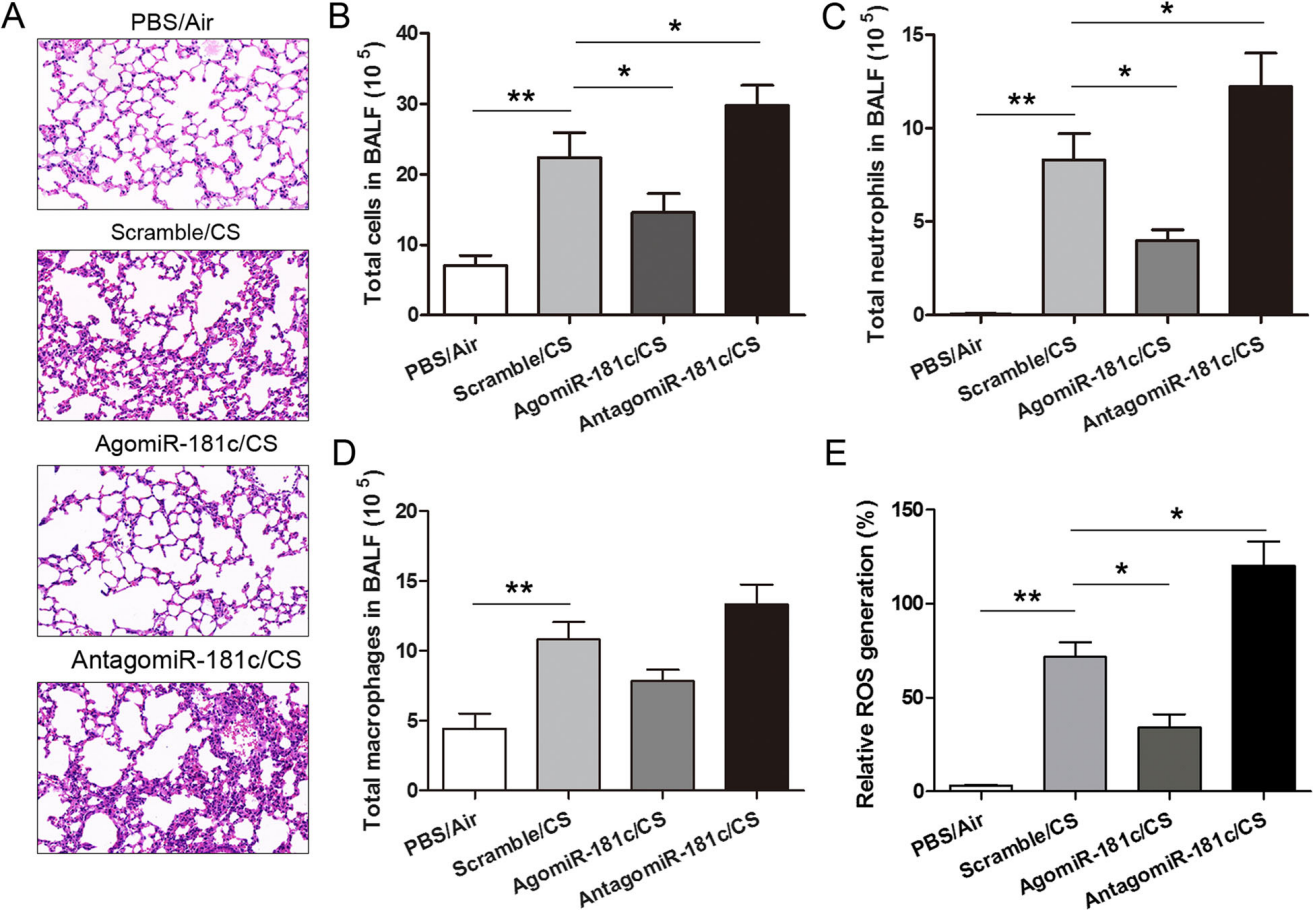
miR-181c inhibits lung injury, neutrophil infiltration, and ROS generation. **a** The representative H&E-stained micrographs of lung tissue from the PBS/Air, PBS/CS, agomiR-181c/CS, and antagomiR-181c/CS group animals at 24 weeks (×20). The total cell number **b**, number of neutrophils **c**, and number of macrophages (**d**) were detected in the BALF of mice in the different groups at 24 weeks. **e** ROS production by BECs of mice in the different groups at 24 weeks was determined as described. Data are presented as means ± SD (*n* = 6 per group). *p < 0.05, **p < 0.01

To investigate the biological function of miR-181c in COPD, we performed in vivo miR-181c over-expression or knockdown experiments using agomiR-181c or antagomiR-181c through intranasal administration. As shown in Fig. 2a, in the CS-exposed mice, degeneration of lung tissue, necrosis, and neutrophil infiltration were increased after 24 weeks of CS exposure as compared with the control group. Over-expression of miR-181c impeded CS-induced lung injury, while inhibition of miR-181c enhanced lung injury. Given that immune cells, such as neutrophils and macrophages, play crucial roles during the process of COPD [17], we next investigated the role of miR-181c in immune cells during COPD. As shown in Fig. 2b and Fig. 2c, the total cells and neutrophils in the bronchoalveolar lavage fluid (BALF) of the agomiR-181c group were significantly decreased, but were increased in the antagomiR-181c group as compared with the CS-exposure group. The numbers of macrophages in the BALF of CS-exposure group were significantly increased, but were not statistically significant in the agomiR-181c group and antagomiR-181c group as compared with CS-exposure group (Fig. 2d). Furthermore, because oxidative stress, such as ROS, overwhelms endogenous antioxidant systems and plays a crucial role in COPD [18–20], we measured the ROS levels in the different groups. As shown in Fig. 2e, ROS generation was significantly decreased in BECs of the agomiR-181c group as compared to that of CS-exposure group. When miR-181c was inhibited, ROS generation increased significantly, clearly indicating an important role of miR-181c in ROS generation during COPD.

### Effect of miR-181c on inflammatory cytokine levels in CS-exposed mice and cells

**Fig. 3:**
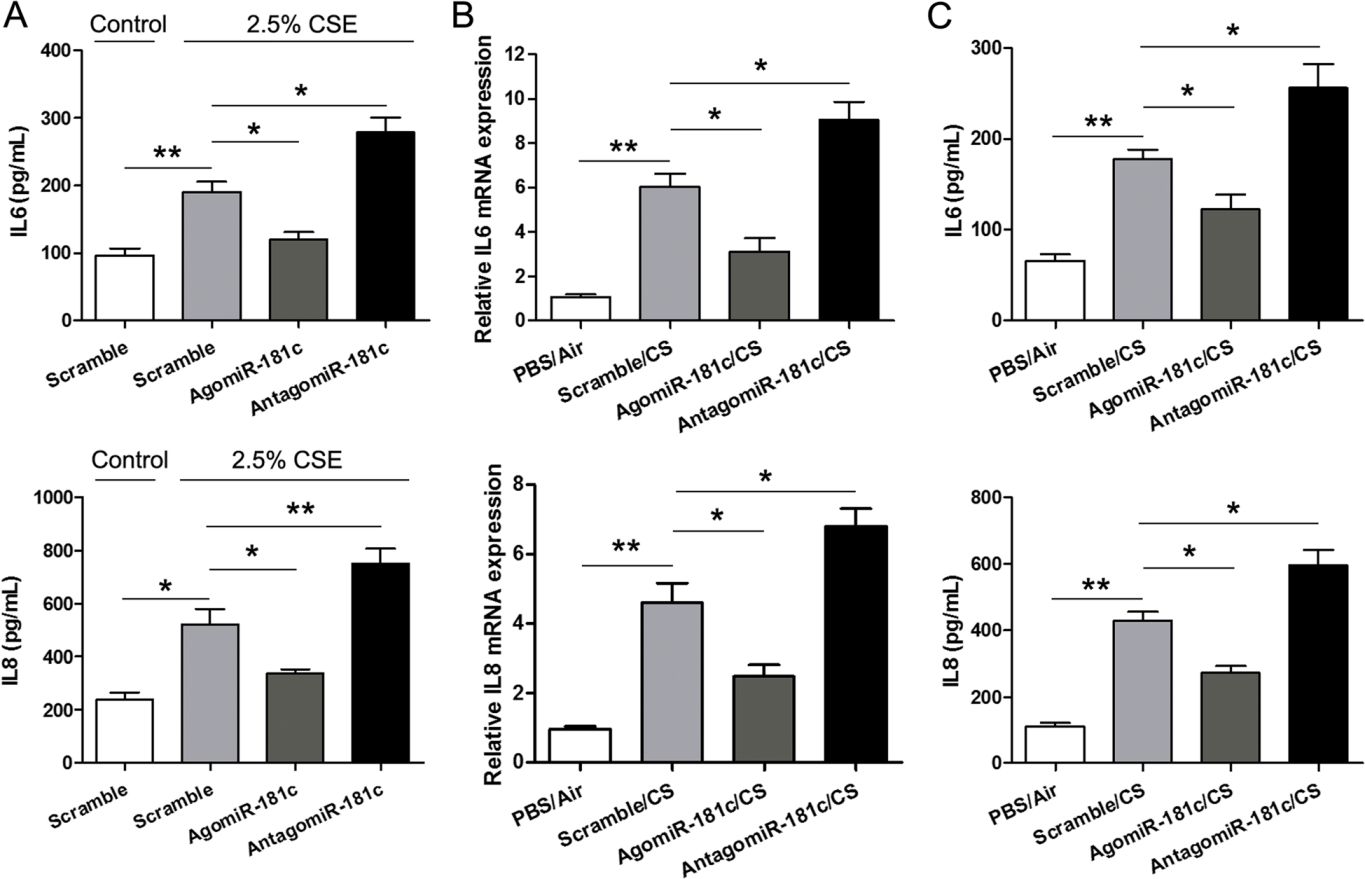
miR-181c decreased the levels of IL-6 and IL-8 in CS-exposed cells and mice. **a** HBECs were transfected with agomiR-181c or antagomiR-181c and then exposed to 2.5% CSE for 24 h. ELISA was performed to analyze IL-6 and IL-8 levels in cell supernatant. The levels of IL-6 and IL-8 mRNA (**b**) and protein (**c**) were measured by qRT-PCR and ELISA in PBS/Air group, scramble/CS group, agomiR-181c/CS group, and antagomiR-181c/CS group animals after 24 weeks. Data are presented as means ± SD (*n* = 6 per group). *p < 0.05, **p < 0.01

Because inflammatory reaction has been shown to be involved in COPD [21], we next determined IL-6 and IL-8 expression in HBECs transfected with agomiR-181c or antagomiR-181c after treatment with 2.5% CSE. As shown in Fig. 3a, CSE promoted both IL6 and IL8 levels compared with the control group; IL6 and IL8 levels were lower in cells transfected with agomiR-181c, but higher in cells transfected with antagomiR-181c, compared to the scramble controls. Furthermore, levels of IL-6 and IL-8 mRNA and protein expression were increased in lung tissue homogenates from CS-exposed mice compared to air-exposed controls; agomiR-181c apparently reduced levels of IL-6 and IL-8, whereas antagomiR-181c increased IL-6 and IL-8 levels compared with the scramble group (Fig. 3b and c). These results indicated that miR-181c inhibited the inflammatory response in CS-exposed cells and mice.

### miR-181c exerts its effect via negatively regulating CCN1 expression in COPD

**Fig. 4:**
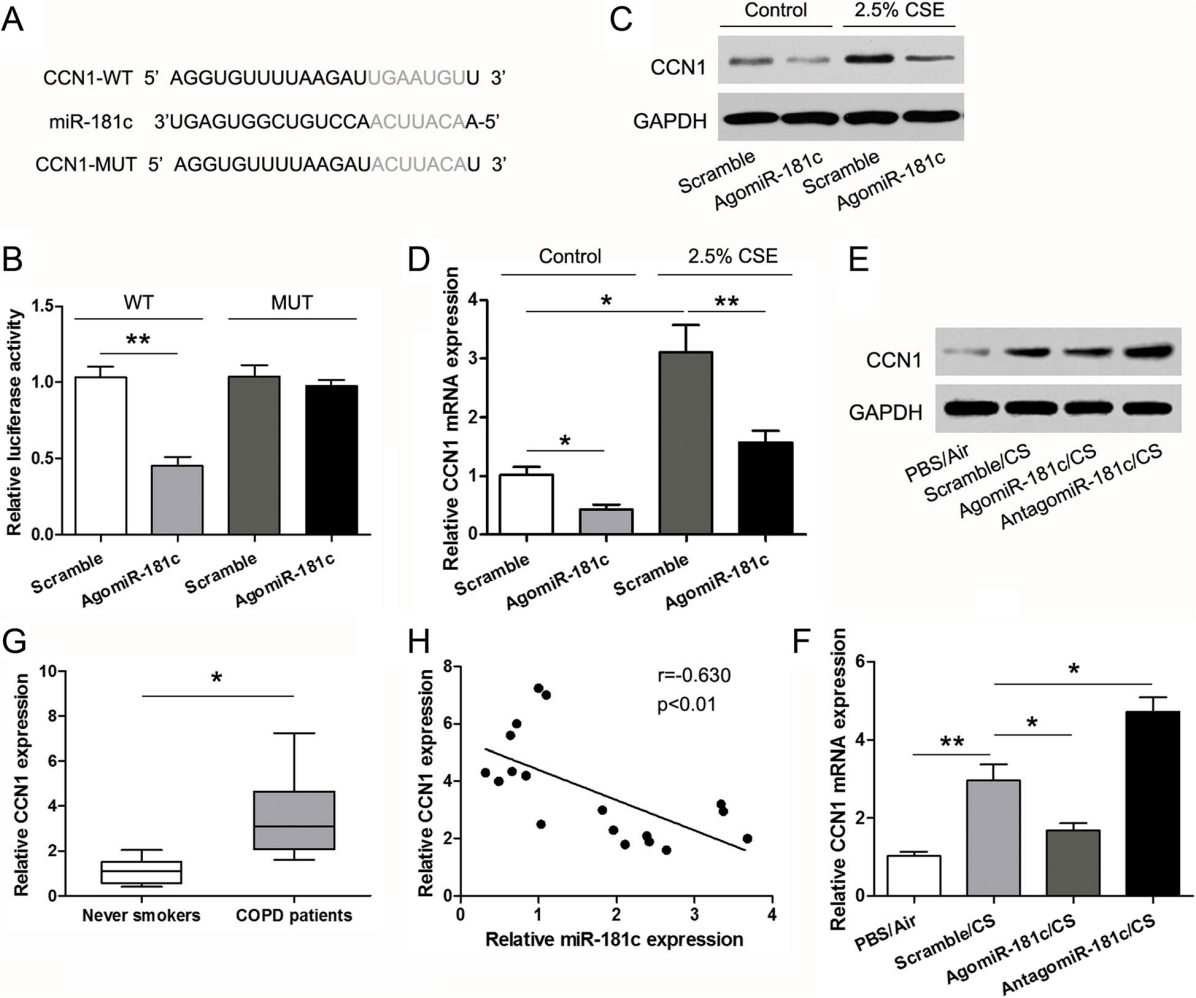
miR-181c negatively regulates the expression of CCN1 by directly targeting the CCN1–3′-UTR. **a** miR-181c binding sites in the CCN1 3′-UTR. Mutations in the complementary site for the seed region of miR-181c in 3′-UTR of CCN1 gene are indicated. **b** Wild-type or mutant reporter plasmids were cotransfected with agomiR-181c or antagomiR-181c into 293 T cells. After 48 h transfection, luciferase activity was determined. Western blot (**c**) and qRT-PCR (**d**) analyses of the expression of CCN1 in HBECs exposed to 2.5% CSE or control medium and transfected with agomiR-181c or scramble. Western blot (**e**) and qRT-PCR (**f**) analyses of CCN1 expression in lung tissues of mice treated with PBS/Air, CS, agomiR-181c/CS, and antagomiR-181c/CS. **g** The relative expression levels of CCN1 were examined by qRT-PCR in lung tissues of 8 never smokers and 18 COPD patients. **h** Pearson correlation analyses between miR-181c levels and mRNA expression levels of CCN1 in human COPD tissues. Data represent mean ± SD from three independent experiments; *p < 0.05, **p < 0.01

To elucidate the underlying molecular mechanisms through which miR-181c exerts its effect in COPD, we used two publicly available bioinformatic algorithms, TargetScan and miRanda, to identify potential target genes regulated by miR-181c. CCN1, also named Cyr61, was predicted to harbor one highly conservative miR-181c binding site in the 3′-UTR of CCN1 at position 519–525 (Fig. 4a). To verify that CCN1 is a direct target of miR-181c, we cloned a reporter plasmid containing the wild-type (WT) or mutant (MUT) 3′-UTR of CCN1. Co-transfection of agomiR-181c and CCN1–3′-UTR-WT strongly decreased luciferase activity, whereas co-transfection of agomiR-181c and CCN1–3′-UTR-MUT did not alter luciferase activity (Fig. 4b), indicating that miR-181c can bind to the CCN1–3′-UTR. Furthermore, western blot and qRT-PCR analyses showed that agomiR-181c suppressed CCN1 expression in HBECs (Fig. 4c and d). In animal experiments, CCN1 expression was increased in lung tissues from CS-exposed mice compared to air-exposed controls; agomiR-181c suppressed CCN1 expression, whereas antagomiR-181c enhanced CCN1 expression compared with the scramble group (Fig. 4e and f). We further examined the expression levels of CCN1 in 6 never smokers and 18 COPD patients. Compared with never smokers, the relative expression levels of CCN1 were significantly increased in lung tissues of COPD patients (*p* < 0.01; Fig. 4g). In addition, correlation analyses revealed that miR-181c levels were negatively correlated with expression levels of CCN1 in human COPD tissues (Fig. 4h). Taken together, these findings demonstrated that miR-181c may exert its effect through regulating CCN1 expression in COPD.

## Discussion

The development of COPD often involves various comorbidities, making COPD a major cause of mortality worldwide, seriously threatening the quality of human life [22]. Emerging evidence has shown that miRNAs play essential roles in COPD [6]. However, the role of miR-181c in COPD remains unclear. In our study, we explored the biological activity of miR-181c in COPD. Functional assays demonstrated that miR-181c over-expression alleviated and miR-181c inhibition aggravated lung injury in COPD. Subsequent investigation revealed that CCN1 is the direct and functional target of miR-181c in COPD. Taken together, our data suggest the critical roles of miR-181c and its target CCN1 in COPD development, and provide potential therapeutic targets for COPD treatment.

Previous studies have shown that miR-181c is implicated in regulation of the inflammatory response, energy metabolism, and cancer development. Fang and colleagues showed that miR-181c targeting TRIM2 ameliorates cognitive impairment induced by chronic cerebral hypoperfusion in rats [23]. Wang and colleagues reported that miR-181c targets Bcl-2 and regulates mitochondrial morphology in myocardial cells [24]. Yang et al. recently reported that miR-181c limits nitration stress of endothelial cells in diabetic db/db mice through inhibiting the expression of FoxO1 [25]. Here, we showed that the expression of miR-181c was decreased significantly in COPD clinical samples. Over-expression of miR-181c alleviated lung injury and neutrophil infiltration in CS-exposed mice, whereas miR-181c inhibition had the opposite effect. In addition, ROS generation was markedly increased in CS-exposed mice, and miR-181c over-expression reduced ROS generation, indicating that miR-181c decreased ROS generation in COPD. Moreover, levels of IL-6 and IL-8 in lung tissues were increased in CS-exposed mice; miR-181c over-expression reduced levels of IL-6 and IL-8, demonstrating that miR-181c can suppress the inflammatory response in COPD.

CCN1, also known as Cyr61, GIG1, or IGFBP10, belongs to the CCN protein family. It is expressed in a broad range of cells, such as fibroblast, osteoblast, endothelial cells and lung epithelial cells [26, 27]. Previous reports showed that CCN1 promotes the adhesion of endothelial cells, interacts with several integrins and with heparan sulfate proteoglycan, and plays a role in cell proliferation, differentiation, angiogenesis, apoptosis, and extracellular matrix formation [28]. Notably, CCN1 is likely a central signal dispatcher controlling the direction of lung pathogenesis, such as inflammation, apoptosis and fibrosis. Several reports have shown important roles of CCN1 in lung epithelial cell apoptosis after oxidative stress [29]. Moon and colleagues investigated the molecular and cellular mechanisms by which CSE triggers IL-8 release and found that CCN1 expression was up-regulated in lung epithelial cells by CSE via induction of ROS and endoplasmic reticulum stress, which further resulted in augmented IL-8 release through activation of the Wnt pathway [27]. In the present study, CCN1 was found to be the direct and functional target of miR-181c. Moreover, miR-181c levels were negatively correlated with expression levels of CCN1 in human COPD samples. These results suggest the critical roles of miR-181c and its target CCN1 in COPD development, and provide potential therapeutic targets for COPD treatment.

## Conclusion

Collectively, all data from our study demonstrate that miR-181c over-expression alleviated lung injury in COPD, as evident from the resulting amelioration of lung injury, reduction of the inflammatory response, neutrophil infiltration, and ROS generation, and down-regulation of CCN1 expression. Taken together, our data suggest the critical roles of miR-181c and its target CCN1 in COPD development, and provide potential therapeutic targets for COPD treatment.

## Abbreviations

BALF: Bronchoalveolar lavage fluid
COPD: Chronic obstructive pulmonary disease
CS: Cigarette smoke
CSE: Cigarette smoke extract
HBECs: Human bronchial epithelial cells
miR-181c: microRNA-181c
qRT-PCR: Quantitative real-time PCR
ROS: Reactive oxygen species
